# A Spiking Network Model of Decision Making Employing Rewarded STDP

**DOI:** 10.1371/journal.pone.0090821

**Published:** 2014-03-14

**Authors:** Steven Skorheim, Peter Lonjers, Maxim Bazhenov

**Affiliations:** Department of Cell Biology and Neuroscience, University of California Riverside, Riverside, California, United States of America; University of Iowa, United States of America

## Abstract

Reward-modulated spike timing dependent plasticity (STDP) combines unsupervised STDP with a reinforcement signal that modulates synaptic changes. It was proposed as a learning rule capable of solving the distal reward problem in reinforcement learning. Nonetheless, performance and limitations of this learning mechanism have yet to be tested for its ability to solve biological problems. In our work, rewarded STDP was implemented to model foraging behavior in a simulated environment. Over the course of training the network of spiking neurons developed the capability of producing highly successful decision-making. The network performance remained stable even after significant perturbations of synaptic structure. Rewarded STDP alone was insufficient to learn effective decision making due to the difficulty maintaining homeostatic equilibrium of synaptic weights and the development of local performance maxima. Our study predicts that successful learning requires stabilizing mechanisms that allow neurons to balance their input and output synapses as well as synaptic noise.

## Introduction

The purpose of building neural networks can be seen from two different perspectives. From an experimentalist's point of view they can be used to help find, validate, or falsify mechanistic theories about the brain through comparison with experimental data. From an engineering perspective they are powerful algorithms to solve computational problems. These perspectives are complementary. Specifically, biological neural networks (e.g. human and animal brains) can solve complex problems; therefore, a properly designed and valid biological model must also be able to solve complex problems. Currently, however, validation through problem solving is rare. Typically brain models are only validated by comparison with experimental data. One of the reasons is because there is no guarantee that even a model consistent with experiments is developed sufficiently for problem solving. In our work however we have chosen to concentrate on problem solving as a validation tool for showing the capabilities and drawbacks of rewarded spike timing dependent plasticity (STDP) in biologically inspired spiking neural networks.

Reward-modulated STDP was proposed as a learning rule capable of solving the distal reward problem in reinforcement learning [1], [2], [3], [4].The distal reward problem [5] arises because the mechanisms of reinforcement learning must be dependent on both the network activity and a reward signal. In any biological organism, the reward is often not received until several seconds after the activity that resulted in the correct response. When reward signal arrives, the relevant activity has long since subsided and the relevant neurons and connections may well have been involved in other activities during this period. This leads to the question of how the problem of correct linking synaptic activity and the behavioral reward is solved in the animal or human brain. Rewarded spike time dependent plasticity is proposed as a solution to this problem. It has been hypothesized that spike time dependent traces are created and in some way stored at a synaptic terminal whenever the pre and post synaptic neuron both experience firing events [6], [7]. When these traces are later reinforced by a reward signal (often believed to be dopamine [8], [9], [10]), they create long-term changes in synaptic strength. These earlier theoretical studies have recently been supported by data from insects [11].

Different classes of learning rules have been developed to address the distal reward problem [12]. Earlier studies, however, are mainly focused on conceptual proof that rewarded STDP has the potential of solving the problem of linking synaptic traces and reward signal. These often use problems requiring only one or two learned outputs. Minimal effort has been deployed to show whether rewarded STDP alone can be sufficient to solve a biologically relevant problem requiring accurate decision making in an uncertain environment or what additional constraints are necessary to make this mechanism operational.

In this new study, we use a multi-layer network of realistic spiking neurons representing a basic biological circuit to solve a complex and biologically relevant problem. Specifically we constructed a decision making network of excitatory and inhibitory neurons, modeled as a virtual entity foraging in a simulated environment. The network uses rewarded STDP to learn the foraging task. Then we examined the limitations of its ability to learn a correct decision-making under a variety of network designs and environmental conditions.

## Results

### Network performance in the random virtual environment

**Figure 1:**
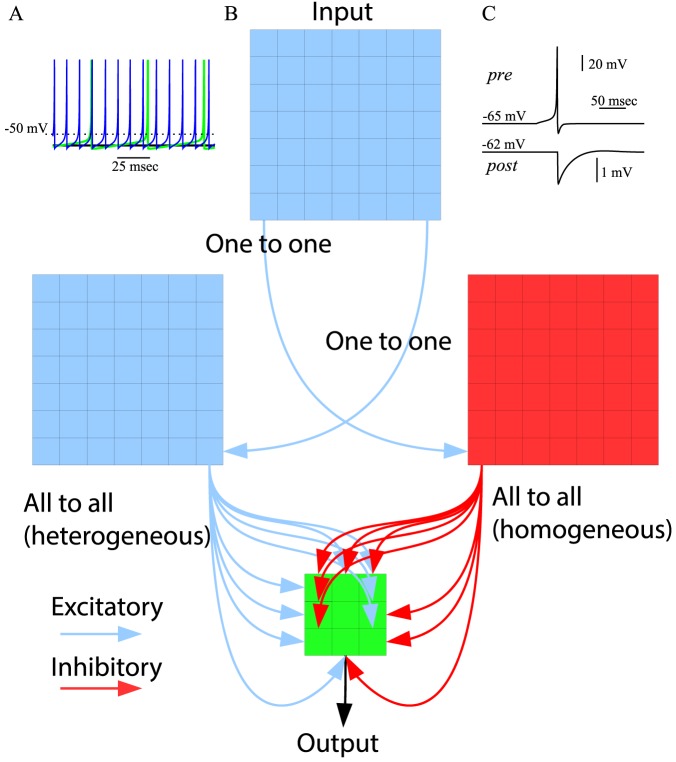
Model properties. (A) Steady-state response pattern of an isolated spiking neuron for three different levels of the resting potential: black – 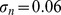, green –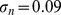, blue – 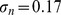. (B) Network organization. Arrowed lines indicate outgoing connections of a sample of cells in each layer with excitatory cells shown in blue, inhibitory cells shown in red and output cells shown in green. (C) Sample IPSP in the postsynaptic neuron (bottom trace) triggered by a spike in presynaptic inhibitory neurons (top trace).

**Figure 2:**
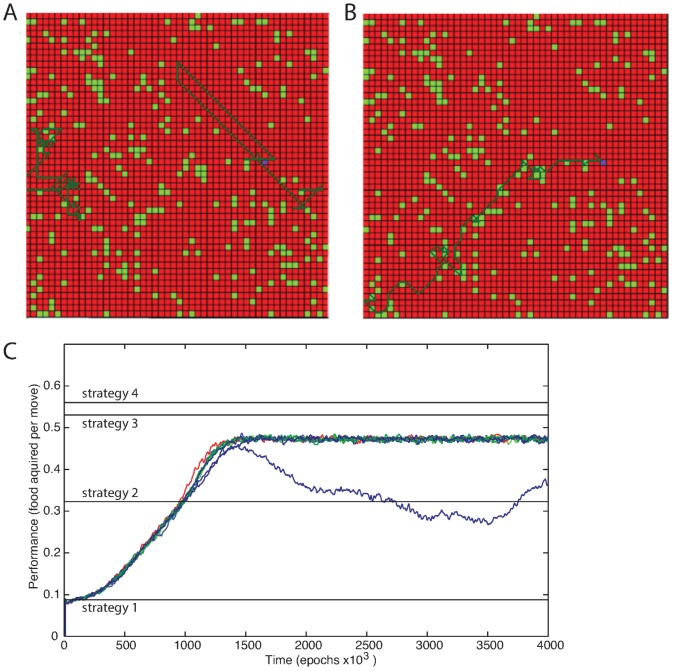
The change in rate of “food” acquisition as a result of learning. (A, B) Trajectory of the movement in the virtual environment. (A) Before training. (B) After training (one million iterations). Light green dots represent “food” location. Red dots are locations without food. Dark green line traces the entire movement. (C) Performance for 6 independent trials (different colors) over 4 million iterations. One of the trials (blue line) failed to achieve normal rates of performance. Horizontal lines represent constant performance of other strategies in solving the same problem. 1 - blind strategy; 2 – collecting adjacent food; 3 - moving towards the closest “food” within three grid squares; 4 - searching through all possible sets of moves within the visual field.

The model included three layers of spiking neurons (Fig. 1A) connected with chemical synapses (see Fig. 1C for example of inhibitory response); the middle layer included populations of excitatory and inhibitory neurons to provide feedforward inhibition to the neurons of the output layer (Fig. 1B). The input to the system was presented as a 7×7 “visual field” represented by the input layer; “food” particles corresponded to depolarizing current that was applied to the corresponding neuron in the 7×7 input layer. Direction of movement was controlled by 3×3 output array. At the onset of the simulation all synaptic weights to the output layer were of uniform strength. In this condition, output layer spikes only occurred due to random variation in the output of individual synaptic events from the middle layer to the output layer. As a result the virtual entity using default settings initially moved primarily along a straight paths with occasional random turns (Fig. 2A).

On occasion an output spike was generated which resulted in movement which lead to successful “food acquisition”. When this event occurred, the network was rewarded and the recently active synapses associated with this response were strengthened. This increased probability of correct (toward food) movement in successive iterations. Over the course of the simulation the virtual entity learned not only to respond to input signaling the position of adjacent “food” but to more distant “food” as well. In general, once trained, the virtual entity was attracted toward higher concentrations of “food” with a bias toward “food” that is closer (Fig. 2C).

To quantify performance of the model we used an exponential moving average that continually approaches the rate of “food” acquisition. It is defined by the equation
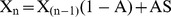
where *X_(n)_* is the performance score at the time of the current move, *X_(n−1)_* is the performance score at the time of the previous move, *S* = 1 if “food” was obtained at this move and *S* = 0 otherwise, *A* is an arbitrary positive constant, *A*<<1. The value used in these simulations was *A* = 0.00001. Qualitatively this expression gives a value that is continuously approaching the current rate of “food” acquisition per move.

It is helpful to compare this performance to other possible strategies for solving the given foraging problem. Four strategies were used to make this comparison (Fig. 2C; see methods); none of these strategies involved learning, the system's behavior was preprogrammed according to a particular strategy. Strategy 1 was a blind strategy, moving in straight lines with occasional random turns. Strategy 2 always collected adjacent “food” if available otherwise it moved according to strategy 1. Strategy 3 moved towards the closest “food” within three grid squares. Strategy 4 was a strong strategy that searched through all possible sets of moves within its visual field. It then choose the first move of the set of moves which collect the most “food” with a bias toward obtaining “food” sooner. Performance of the virtual entity varied because of inherent noise in the model and the environment it forages in (4 different trials are shown in color in Fig 2C). Usually virtual entities using default model settings reached similar levels of performance, slightly below strategy 3 (see red, green and black lines in Fig 2c). However they occasionally became trapped in local maxima resulting in lower performance (blue line).

Importantly, the networks performance after training does not depend on the specific implementation of the virtual environment used in training phase. The network trained in one environment, still demonstrated high level of performance for any random distribution of the food particles with similar statistical properties. Changing properties of the food distribution, however, led to the overall change in performance (see below).

**Figure 3:**
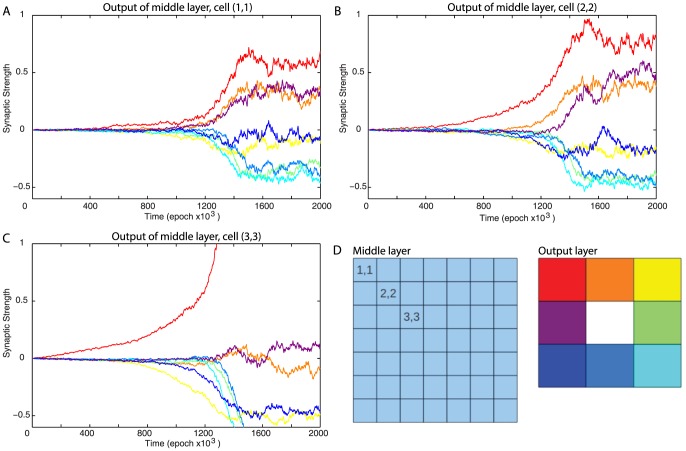
Synaptic weights dynamics during learning. (A, B, C) These plots show the strength of synaptic outputs of three different cells over the course of the experiment. Values shown are relative to the mean weight of out put synapses of the cell. Each graph shows the synapses from one middle layer cell to each of the 9 output cells. The synapses are color-coded based on which output cell they connect to. Synaptic values are truncated within the range [−0.6, +1]. (D) Schematic location of the 3 cells shown in panels A–C (left) and color-coding of output cells (right).

To evaluate synaptic changes induced by learning, we analyzed the dynamics of synaptic weights. Fig. 3 A–C shows the evolution of the outgoing synaptic weights of three middle layer cells that were located in the upper/left direction from the center of the layer (Fig 3D). These cells represented successive cells in the top/left area of the “visual field” and sent connections to each cell in the output layer. The synapse from the upper/left cell that was closest to the center of the middle layer (cell (3,3)) to the top left output cell (red trace) increased in strength as responses connecting activation of this middle layer cell, which represented the adjacent area in the upper/left direction, and movement in the upper/left direction were the most likely to be rewarded (Fig 3C). (Note, that this weight saturated at ∼5 and was truncated in the Fig. 3C to allow sufficient resolution of other traces). Over time, however, synapses to output cells which moved the virtual entity up and left (orange and purple traces) were also strengthened as responding to activation of these mid layer cells by moving in these directions was more likely to move the virtual entity toward “food” than away from it. These lower strength connections allowed the network to integrate information from many input cells. The network behavior and direction of movement selected depended upon the input from multiple cells. The network was observed to respond to higher concentrations of food rather than responding reliably to food in individual locations. The synaptic strength of outgoing synapses of other middle layer cells located further from the center (cells (1,1) and (2,2)) stabilized at less extreme values as there was a weaker correlation between a given response and a reward. This gave them weaker influence over the direction of the movement. Finally, synapses connecting middle layer cells in the top/left area of the visual field to the bottom/right output cells (e.g., yellow trace) decreased their strength, as they were least likely to trigger movement to the right direction.

### Effect of model changes on the network performance

**Figure 4:**
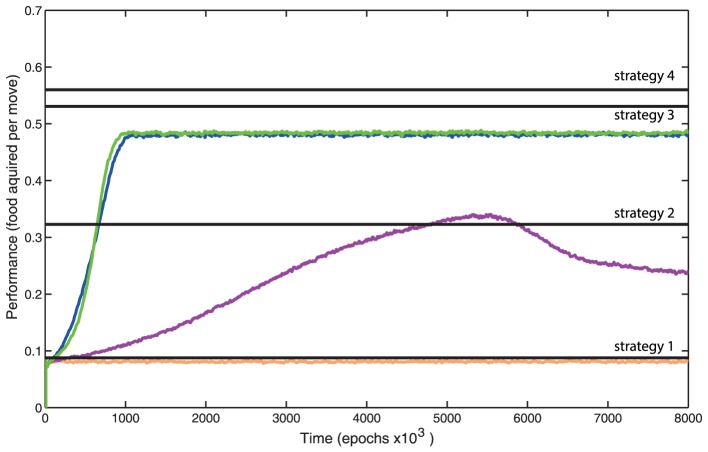
Performance after elimination of different model features over 8 million movement itterations. Each line corresponds to performance after removing one feature. Green is default. Blue corresponds to the network when punishment was turned off. Magenta shows a network with no output balancing. Orange represents a network with no variability in synaptic release.

To evaluate the role of different mechanisms in the overall learning performance, we systematically turned them off one by one (Fig. 4). In each experiment one major feature of the model was removed and its performance over time was plotted. Baseline model performance was represented by green trace. In the first experiment (Fig. 4, blue line) the punishment mechanism was turned off, the punishment mechanism applied the inverse and reduced value of the currently active STDP traces (see Methods, eq (3)). The network still received reward when “food” was obtained but no change occurred when “food” is not obtained. Learning rate is reduced slightly but no other changes were observed. The second experiment (Fig. 4, magenta) explored a network that did not make use of output balancing. Output balancing reduced the rate at which outputs were strengthened by reward when the neuron had a large sum of output strengths (see Methods, eq (4)) so the rate at which outputs were strengthened was no longer dependent on the total output strength of the presynaptic cell. This resulted in low and unstable performance, though the performance was still better than random motion (Strategy 1 from fig 2C). In the third experiment (Fig. 4, orange), variability in synaptic release was eliminated. Under this condition the depolarization applied to the cell was always directly proportional to the strength of the synapse. This resulted in no activity in the output cells and consequently no learning. The virtual entity moved in a straight line with a very low probability (*p = 0.02*) to turn in a random direction. These random turns were explicitly implemented to the model and present in all conditions (see Methods). The output cells did not fire because the amount of inhibition and excitation to a given output cell were equal in magnitude. Finally, in the fourth experiment (not shown), input balancing was removed such than the total incoming synaptic strength to a cell was allowed to change when STDP traces were rewarded. Without this homeostatic mechanism, the sum of the input strengths to the output cells either fell very low or became very high. Indeed, when positive STDP events were rewarded the temporal correlation between pre and postsynaptic activity became stronger. This increased the likelihood of further potentiating events. This led to runaway synaptic dynamics and the network quickly became unstable and the virtual entity moved in random or repetitive circles until the network far exceeded physiological range of synaptic changes.

We also tested a canonical simplified version of STDP alone and found that it was not sufficient to perform the successful learning of the presented task. Without balancing of the input synaptic connections (eq (6)), some synaptic weights continued to grow leading to unstable dynamics; with explicit limits implied to the maximal weight we still observed run away synaptic dynamics leading to bimodal distribution of synaptic weights and very low model performance (similar to the orange trace in figure 4).

**Figure 5:**
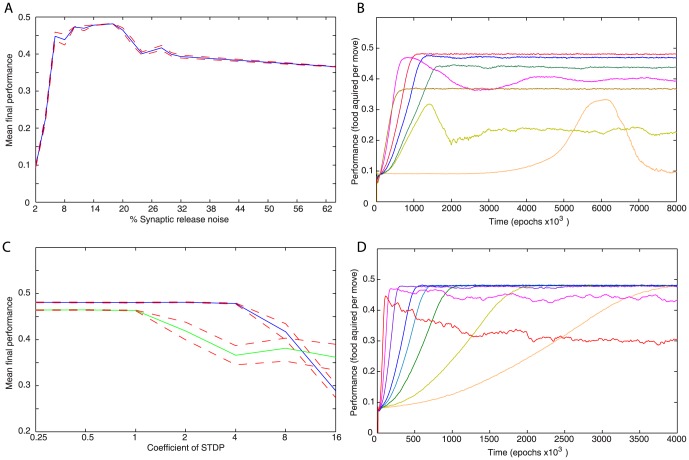
Effect of noise and STDP strength on learning performance. STDP strength is scale in equation 1 from the methods section. (A) Plot of mean final performance with variable levels of variability in synaptic release. Twenty-five simulations were run under each noise condition and final performance was recorded after 4 million moves. Red dashed lines shows the limits of standard error. (B) Plot of mean performance over time with variable levels of variability in synaptic release represented by different lines. Twenty-five simulations were run under each noise condition over 8 million moves. Noise level: 2% orange, 4% gold, 8% dark green, 12% blue, 16% red, 32% magenta, 64% brown. (C) Plot of mean final performance with variable STDP coefficient strength. Twenty-five simulations were run under each STDP coefficient condition and final performance was recorded after 8 million moves. Two sets were run with different noise levels: 16% release noise is shown in blue and 8% is shown in green. Red lines show standard error. (D) Plot of mean performance over time for different STDP strength. Twenty-five simulations were run for each STDP strength over 8 million moves (4 million shown). Release noise is set to 16%. STDP strength: orange-0.25; gold-0.5; dark green-1; light blue-1.5; dark blue-2; purple-4; magenta-8; red-16.

We found that synaptic noise was critical to achieve high model performance. Figures 5A,B show data corresponding to a series of simulations where the level of random variability in synaptic release (R from equation 8 in the methods section) was varied between 02 and 64. The final performance was maximized with noise levels between 08 and 16 but dropped off at higher or lower levels of noise (Fig. 5A), however it remained relatively high even for high levels of noise. Furthermore, we found some trade off between final performance and learning speed related to the level of noise (Fig. 5B). Higher noise levels continued to improve learning speed even though they resulted in the lower final performance.

Figures 5 C,D contain data corresponding to a series of simulations where the STDP strength (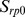 from equation 4 in the methods section) was varied across a wide range, altering the rate at which synapses could change. Numbers shown are relative to a default of 1. From figure 5C it can be seen that final performance is maximized with lower STDP coefficient strengths. This is expected because it allows the network to more finely tune synaptic strengths. We also see that at higher levels of synaptic noise, the network became greatly more tolerant of higher rates of STDP coefficients. Figure 5D, however, shows a trade off between final performance and learning speed as the rate of STDP changed. Higher STDP coefficients led to faster learning but at very high values the final performance was affected.

### Effect of environmental changes on the network performance

**Figure 6:**
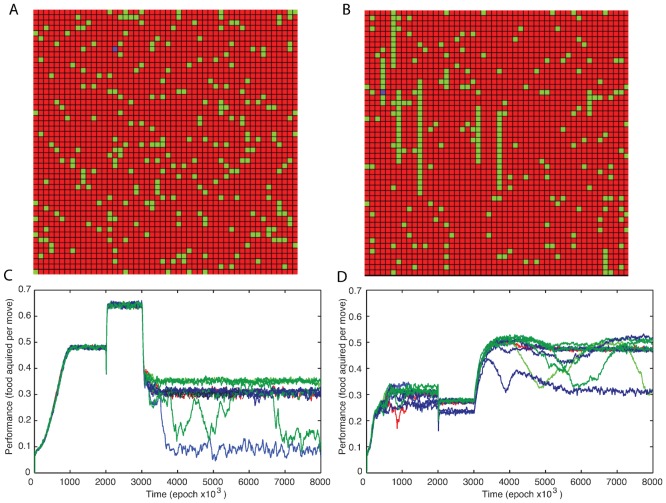
Effect of changing the environment. (A) Normal “food” distribution. (B) A vertically biased “food” distribution. (C) Performance over time of the network starting in a normal environment then being switched to a vertically biased environment at 2,000,000 iterations. Learning was turned off and all synaptic weights were held constant until 3,000,000 epochs when learning was turned on again. (D) Performance over time of the network starting in a vertically biased environment then being switched to a normal environment at 2,000,000 epochs. Learning was turned off and all synaptic weights were held constant until 3,000,000 epochs when learning was turned on again.

Next we studied change in the model performance following changes in the “food” environment. Since the model learned the statistical properties of the food distribution and not a specific pattern of the food particles, changing the random environment to another one characterized by similar statistics of food distribution did not affect performance of the trained model (not shown). Therefore, we explored the effect of changing the random environment to a different one that was biased toward a particular pattern of food particles. In the first experiment virtual entity was initially trained on a normal, random distribution (Fig 6A) and the environment was then changed to a vertically biased distribution (Fig 6B) at the midpoint of the experiment (at time 2,000,000, Fig 6C). At this time learning was turned off. The vertically biased environment was created by biasing food placement in favor of placing “food” directly above or below existing food. This tended to arrange “food” into vertical columns.

**Figure 7:**
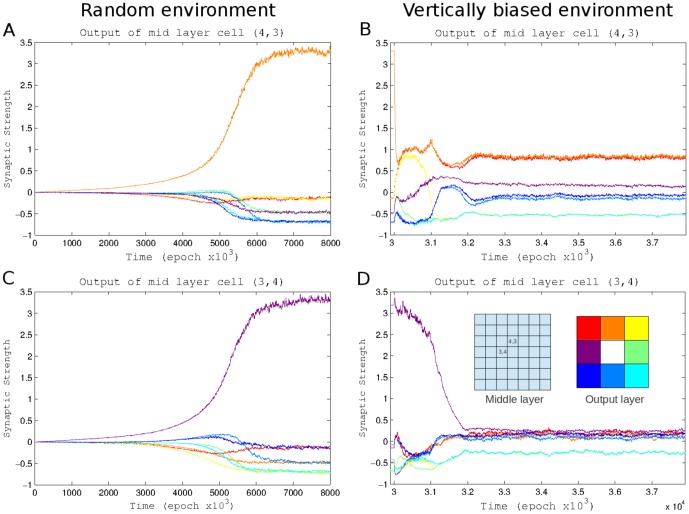
Effect of changing environment on synaptic strength. (A) Synaptic strengths of the outputs of a middle layer cell to all output cells during learning under normal conditions. This cell indicates food immediately above of the entity. (B) Synaptic strengths of the same cell after environment was later changed to a vertical arrangement. (C and D) Same as A and B but for a cell indicating food immediately to the left. (E) Shows the location of cells in the middle layer and the color representation of outputs by destination cell.

The network training in the normal environment allowed it to be very successful in the new environment (Fig 6C). It was even more successful in the vertically biased environment than it was in its normal environment as this arrangement of the “food” was more likely to have clusters of connected food. When learning was turned back (at time 3,000,000), the simulation performance was rapidly reduced. When food was arranged vertically food was more likely to be located in the upward or downward direction from any position where the entity acquired food. Connections involved in acquiring “food” above or below the entities current location were more likely to receive reward than those that indicated any other direction. The result of this was a sharp decrease in synaptic strength of the synapses involved in movement toward food in other directions (compare magenta traces in Fig. 7 C and D). Counter intuitively we observe the connections involved in obtaining food in the vertical directions decrease as well, even though they remained augmented enough to promote “correct” movements (compare orange traces in Fig. 7 A and B). Since multiple food particles were likely to be found above or below current location, connections promoting moving Up or Down (such as orange trace in Fig. 7B and similar connections from middle layer cells (4,2) and (4,1)) together triggered fast spiking response of the output cell responsible for Up/Down directions and were constantly rewarded. However, any other connection that was (by chance) strong enough to mediate output cell firing (such as red trace in Fig. 7B) was also rewarded even though it did not control direction of movement (because “red” cell firing was delayed compare with “orange” output cell firing). In result these connections remained high and the output weight balancing (see Methods) prevented Up/Down connections from further increase.

This could be seen as similar to repetitive motions observed in motor stereotypies. Although the model could continue to obtain “food” when the “food” was directly above or below it, it was much less capable of dealing with other situations when there was no “food” adjacent to it in these directions.

In the second experiment (Fig 6D) the network was initially trained in the vertically biased environment. It reached lower maximum performance than the networks trained in a random environment achieved under either environmental condition. When the environment was changed to the random distribution and learning was frozen (at time 2,000,000), performance was further reduced. Here again we saw that when a small number of responses regularly resulted in the majority of the rewards received, performance was negatively affected. Turning training back again (at time 3,000,000) led to improvement in performance. Randomly a few implementations of the network that had initially learned under the vertical condition did exceptionally well when the food placement was returned to random (Fig. 6D). A few of these networks showed slightly higher performance than any network that has been observed which learned under the standard random food placement condition (Fig. 6C).

Outgoing synaptic strengths were plotted during the transition from random to vertically biased food placement for two middle layer cells, one indicating adjacent food above the entity (Fig. 7B), and another indicating food to the left of the entity (Fig. 7D). While the strongest connection of both cells decreased rapidly after the change in environment the strong connection of the north-indicating cell retained considerably more strength.

### Effect of the random synaptic strength perturbations on network performance

**Figure 8:**
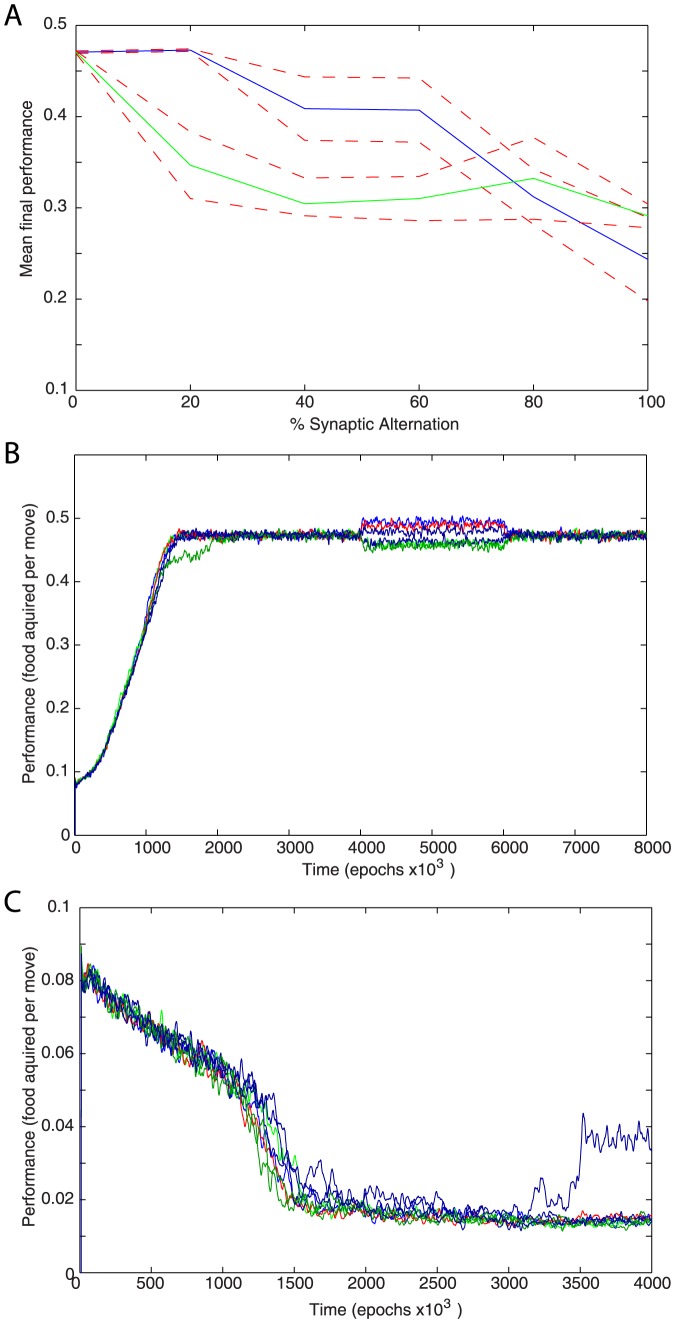
Effect of synaptic noise. (A) Mean final performance of 8 runs with different levels of random perturbation of excitatory synaptic weights from middle layer to output layer. The simulations represented in green applied the perturbation only at the start. Those represented by blue applied perturbations at regular intervals. The thin red lines represent the limits of standard error. (B) 50% random variations applied to synaptic weights of the trained network. Learning was turned off and synapses were held at a fixed strength from 4,000,000 to 6,000,000 iterations. (C) Performance when reward and punishment conditions are reversed in an attempt to train avoidance behavior. Performance in “food” acquisition falls well below random (indicating successful learning) but the model failed to explicitly avoid all food.

In the standard starting condition of the network, all excitatory synaptic weights from the middle layer to the output layer had the same value. To test effect of the variability in initial weight distribution, these weights were initially randomly varied to observe the effect on performance. This randomization was performed by multiplying each excitatory weight by a random number selected from a flat distribution centered on one (e.g. for 20% variation each synaptic weight was multiplied by a number from 0.8 to 1.2). This represented a change in the initial synaptic strength as opposed to the variability of synaptic release (Fig. 5) that occurs each time the presynaptic cell fires. Due to the input side balancing mechanisms described previously (see also Methods), the sum total of synaptic inputs to any one cell, and hence to the layer as a whole, was unchanged by this randomization. The average performance in shown as a green line in figure 8A. Each point represents the average of 8 simulations with different initial set of synaptic weights; thin red lines indicate standard error. The maximum performance attained under conditions of high initial randomization was highly variable. The performance was always higher than random motion (strategy 1) and was often similar to the best performance of a network which only responds to “food” in adjacent squares (strategy 2). A sizable minority of simulations, even among those groups with high initial variability, still attained normal performance levels.

In another set of experiments the weights were once again initiated with the same level of variability. In addition, every one million iterations the weights were partially randomized again using the same approach as for initial weights (multiplied by a new number drawn from the same distribution). The results are represented as the blue line on fig. 8A. Surprisingly for moderate levels of variability the repeated random perturbations of synaptic strength rescued many of the simulations from low performing states. It can be reasonably assumed that the added noise helped the network escape from local performance maxima. At the very high levels of variation, however, no benefit of random noise could be seen.

When the same method of randomizing synaptic weights was applied to the trained network that had already achieved high performance levels, there was no observable lasting effect on performance (Fig. 8B). The level of variation used in these experiments was 50%. In many cases such networks experienced a decrement in performance while learning was frozen but took very little time to return to normal once learning mechanisms were restored. In a few rare cases, performance actually improved slightly during the non-learning phase that followed perturbations of synaptic strength. Any improvements vanished once learning was restored. This indicates that the stable solution arrived at through the default set of mechanisms is not optimal even after variability in synaptic release is accounted for. This solution, however, could resist even strong synaptic weight perturbations.

Finally, in an attempt to train the network to avoid “food” the reward and punishment conditions were reversed (Fig. 8C). The network was rewarded every move in which it did not obtain “food” and punished when it did. Due to the much larger number of empty spaces and the fact that empty spaces are not removed when moved to, this represented a much easier problem. The model was successful in avoiding “food” but did not explore the entire space. It is still worth noting that no other changes were necessary for the network to perform well under these new conditions.

## Discussion

In this study we implemented rewarded STDP to a biologically inspired spiking network model representing a basic neuronal circuit with feed forward excitatory and inhibitory projections. We then asked whether such network is capable of solving a task of learning to map correctly and optimally a multidimensional input space (represented by the patterns of activity of the input neurons) to the multidimensional output space (represented by the output neurons). The learning task was formalized in the context of the basic foraging behavior in a simulated environment of randomly distributed “food” particles. We showed that rewarded STDP model was sufficient to learn the foraging task only when additional rules controlling balance of synaptic weights were implemented. The canonical simplified version of STDP alone was not sufficient to perform the successful learning of the task presented in this study. Without careful maintenance of synaptic homeostasis, learning mechanisms used in the model cause imbalance in the level of activity in the network and in the relative effectiveness of different components in the network. This was overcome by introducing two basic homeostatic mechanisms. One rebalances the weights of synaptic inputs to a cell whenever a synaptic input is strengthened or weakened to maintain the same total of weights. The other modulates total synaptic input to a cell in the network based on its long-term activity. Furthermore, the rate of synaptic facilitation was inversely proportional to the total synaptic output of a cell – output balancing. Together these mechanisms provided stable activity levels, which allowed for rewarded STDP based learning to occur. We further found that synaptic noise and random perturbations of synaptic connectivity during training phase both required to achieve maximal network performance after training.

Neural networks have been theorized to solve biological problems since Alan Turing's B-machines in the 1940s [13]. Usually, a goal of training in such a network is to solve various types of classification problem [14] for a more detailed overview). Advances in understanding of how biological networks operate led to further developments of these types of models and vastly improved capabilities [15], [16]. However, despite mimicking neuronal networks in many respects, many existing models of decision making use an artificial form of back propagation to enable supervised learning [17]. Biological networks are unlikely to be capable of using this powerful technique, as it requires a form of omniscience of the activity of the entire network that would be very difficult for real cells to gain access to. Instead, different forms of Hebbian plasticity [18], [19] are found in neuronal systems whereby fast, large amplitude [Ca^2+^] increases induce potentiation, but slower and low amplitude Ca^2+^ raises induce depression [20], [21], [22], [23], [24], [25], [26]. Furthermore, artificial network's neurons are not constrained to all or nothing output of a biological spiking neuron and communication between cells is not limited to synaptic interactions [27], [28]. Finally, artificial networks can avoid the distal reward problem because input and reward can be artificially correlated in time.

In attempts to use biologically realistic models validated by problem solving, different classes of Hebbian type learning mechanisms were implemented to the networks of neurons in order to solve a complex pattern matching tasks [3], [15], [29], [30], [31]. However, number of simplifications that were employed in both the model design and the task itself prevent these models from being able to address the question of applicability rewarded STDP concept to biological problem solving. Other models applied complex cells and reward modulated plasticity to approach targets, but used plasticity based only on presynaptic firing rate rather than STDP [32]. In one study of decision making based on the reinforcement mechanisms both reward and punishment were required for successful learning [33].

In this study we built a network of simplified spiking neurons capable of learning to solve a foraging problem using rewarded STDP as a primary learning mechanism. Accomplishing this goal requires overcoming a number of issues not present in earlier models with similar goals [34]. We avoid sharing information between neurons except through synaptic communication and a global reward signal from the network. The foraging problem used in this model provided a more naturalistic setting for learning using a simple neural network. While advantages and limitations of the rewarded STDP as a model of reinforced learning have been demonstrated in previous studies, the main objective of the present work was to create a minimal network model of spiking neurons capable of the stable and scalable learning of the properties of the virtual environment and, after training, mapping the input patterns representing snapshots of this environment to the optimal response patterns. The basic neuronal circuits implemented in our model are found in different brain areas, however we did not attempt to model precisely a specific brain structure. Indeed STDP based plasticity and learning occur in different structures (e.g., hippocampus, neocortex) of very different species (including vertebrates and insects [11]). Therefore, we looked to explore general principles required to accomplish a stable (in respect of synaptic changes) learning of a relatively complex task by biologically inspired neuronal network.

It has been demonstrated previously that rewarded STDP is capable of providing reinforcement learning [1], [35]. What is particularly distinct in our study, however, is the complexity of the input/output mapping. A great deal of complexity emerges when diversity is added in the number of possible inputs and outputs. As the number of input/output possibilities increases new features are required to allow responses to compete against one another. This requires the network to be able to achieve and maintain a broad distribution of synaptic connections and to avoid runaway synaptic dynamics, a common effect of STDP alone [36], [37], [38], [39]. Other studies have used feed forward networks to illicit arbitrarily selected spike trains or population responses but still without needing to produce a wide range of responses depending upon input [2], [3]. While we cannot make a claim (without explicit testing) that none of the previously published models would have been able to perform this task, it seems unlikely as the mechanisms that were first implemented in this study, especially output balancing, were found to be essential for solving this complex task. Indeed, a common scenario that was extensively explored in the literature with biologically inspired networks [29], [30], [31], [40] was that the decision making model requires only one or two outputs and as such synaptic weights that had moved to artificially set maximum or minimum values would represent an acceptable solution to the problem. Among other added difficulties, the solution to our task required that connections the network obtains have stable intermediate values.

Rewarded STDP is homeostatically unbalanced. Several mechanisms suggested to prevent the runaway synaptic dynamics are based on adjustment of STDP learning rules per se. These include weight-dependence, so that weaker synapses potentiate more while stronger synapses express less potentiation, and in the limit even depress [36], [41], [42], and/or precise balancing of STDP rules for potentiation and depression [36], [43], [44], [45], [46], [47], [48], [49]. It was shown rigorously that STDP can lead to stabilization of the mean firing rate of the postsynaptic neuron if the integral of the learning window is negative [48]. However, experimental evidence shows a great variety of the duration and magnitude of STDP windows for potentiation and depression [50], [51], [52], [53], [54].

We found that when the synaptic scaling mechanisms described in the previous studies [55], [56], [57] were applied, the network could maintain the balance of synaptic weights and learn to produce better results than random chance and without explicit alternations of the STDP rules. These mechanisms included synaptic input balancing (eq (6)) and slow homeostatic scaling (eq (5)) (Fig. 4, magenta trace). However more advanced scaling mechanisms were required to achieve much higher levels of performance (Fig 4, green/blue traces). Primarily it was necessary to maintain output balancing (eq (4)) which reduced the rate at which synaptic outputs were strengthened by reward when the neuron had a large sum of all output weights. If the last mechanism was not implemented, performance greatly suffered. Importantly, our proposed synaptic rules of input and output balancing are biologically realistic and represent good targets for experimental searches of learning mechanisms.

Many of the mechanisms proposed in our study have clear analogs to biological mechanisms seen in experiments. Rewarded STDP operates similarly to the way dopamine is proposed to affect learning circuits [8], [10], [58], [59], [60]. Balancing of the strengths of a number of inputs to a single neuron in order to maintain a more constant level of input has been observed in experiments [55], [56]. Indeed, rises of intracellular [Ca^2+^] are not restricted to the activated synapses but take place also at synapses, which were not active during the plasticity induction, e.g. due to bursts of backpropagating action potentials [61], [62]. This [Ca^2+^] increase can lead to plasticity at non-active synapses – heterosynaptic plasticity, often also referred to as non-associative plasticity [63], [64], [65], [66], [67]. Recent study suggested that heterosynaptic plasticity may restrict run-away synaptic dynamics mediated by STDP alone [68]. Furthermore, homeostatic scaling of intrinsic and synaptic properties responsible for adjustment of the firing thresholds in response to cell activity has been well documented in neurons [57].

In our model as in behaving animals reward causes increased probability of repetition of behaviors preceding the reward [69]. This is even true in situations where a single behavior that reward too often can be repeated pathologically. In the model this occurred when the environment was changed to feature primarily vertically arranged food squares. The “over learning” of a small set of responses is also observed in animals when the reward system malfunctions such as motor stereotypies after repeated amphetamine application [70], [71].

We found that even when activity levels are stable the network can still encounter serious performance issues when certain neurons develop many strong outputs. This can result in a small number of neurons controlling activity in a large portion of the output layer. Some outputs of these neurons are beneficial and so all of the activity of these neurons are rewarded at above chance rates. Reducing the rate of gain in synaptic strength resulting from rewarded STDP events prevents this by allowing under represented neurons to more easily compete for representation in the next layer. Competition between multiple outputs of the same neuron, as incorporated into this model, makes intuitive sense but has not been a subject of any great deal of study. Our study predicts that such competition is important in preventing a small number of neurons from dominating the networks activity and suggests that future experiments look for evidence of such mechanisms. There are also other ways to implement such competition, e.g., through lateral inhibition between output neurons [71] found in many biological systems.

Synaptic noise was implemented as variability in the magnitude of each individual synaptic event and was necessary for breaking out of local maxima of synaptic strength and therefore, to allow further increase of performance. This was in agreement with previous results supporting the general idea about importance of synaptic variability and noise [72], [73], [74]. We found some trade off between final performance and learning speed related to the level of noise. Surprisingly, higher noise levels continued to improve learning speed even though they resulted in the lower final performance. In addition repeated partial randomizing (random perturbations) of synaptic weights during training rescued many of the simulations from low performing states.

We found that the key themes that unite the mechanisms necessary for the network to be capable of addressing the tasks presented in this study are synaptic homeostasis and noise. It is crucially important to prevent both over representation and under representation of connections for the network to develop balanced synaptic weights. Without such mechanisms some connections in the network will be reinforced to the point that other inputs cannot meaningfully affect the network's behavior. Synaptic homeostasis including output balancing proposed in this study can accomplish these goals without precise tuning of synaptic rules or balancing the potentiation and depression windows of STDP.

## Conclusion

In this study we evaluated the performance of a rewarded STDP model implemented in a biologically inspired spiking network model representing a basic neuronal circuit. Our study predicted that a balancing of both incoming and outgoing synaptic connections was required to achieve high levels of learning performance. Furthermore, it was observed that performance would not improve without the presence of noise within the system and that the level of noise as represented by variability in synaptic release had a great impact on final performance. In exploring the ways in which variability in synaptic release and learning rate can affect the chances of the model to learn effectively and final performance, our study has observed trade-offs between different mechanisms involved in learning and may guide future experimental studies of decision making phenomena.

## Methods

### Learning model

In this study, rewarded STDP was implemented as part of a spiking network model of excitatory cells and inhibitory interneurons. The network was used to model basic foraging behavior in a simulated organism (referred as “virtual entity” below). The foraging behavior took place in a virtual environment of randomly distributed “food” particles. The environment consists of a grid of locations. Each location either has or does not have food. “Food” was distributed randomly on the 50×50 environment grid. The virtual entity sees a 7 by 7 grid of squares the – “visual field” - centered on its current location. It can move to any adjacent square including diagonally for a total of 8 directions. Time is divided up into epochs of 600 time steps, roughly equivalent to 300 ms. At the start of each epoch the virtual entity receives input corresponding to the locations of nearby food. Each cell of the input layer maps to a grid square within 3 squares of the virtual entities location. Thus 48 of the 49 cells receive input from a unique position relative to the virtual entity. During the middle of the epoch the virtual entity makes one move based on the activity of the output layer. The remainder of the epoch acts as a “cooling off period” to allow neurons to return to the resting state. If the virtual entity moves to a grid square with “food” the “food” is moved from that square to a randomly selected new square.

The network was composed of 156 map based neurons [75], [76] in 4 groups arranged into 3 feed forward layers to mimic a basic biological circuit: a 7 by 7 input layer (I), two 7 by 7 middle (hidden) layers, one excitatory (H) and one inhibitory (HI), and a 3 by 3 output layer (O) (Fig 1B). This structure provides a basic feedforward inhibitory circuit [77] found in many biological structures, e.g, thalamocortical [78], hippocampal [79], olfactory [80], [81]and others [82].

Input cells are stimulated by current injection sufficient to trigger a spike if there is “food” on the grid location the cell is mapped to. Each cell of the input layer (*I_i_*, where *i* is cell index) outputs to one cell in the excitatory middle layer by a synapse with strength *W1_ij_* from *I_i_* to *H_j_* and one cell in the inhibitory middle layer by a synapse with strength *W2_ij_* from *I_i_* to *HI_j_*. This is one to one map, so *W1_ij_>0, W2_ij_>0* only if *i = j* and *W1_ij_ = W2_ij_ = 0* otherwise.

Time is divided into epochs of 600 time steps and is represented by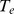. Each epoch is of sufficient duration for the network to receive inputs, produce outputs, and return to a resting state. Input cells receive excitation on the first time step of each epoch. Output is chosen and the virtual entity is moved at the end of the epoch.

Each cell in the excitatory middle layers (cell *H_i_*) or inhibitory middle layer (cell *HI_i_*) connects to every cell in the output layer (*O_j_*) with synaptic strength *W_ij_* or *WI_ij_*, respectably. Initially all these connections have uniform connection strengths (*W_ij_ =  Const, WI_ij_ =  Const* and independent on *i* or *j*). Thus, all responses in the output layer are due to random variability in the activity of middle layer output synapses. This variability is inherent to all synaptic interactions between neurons caused by release noise of synapses. It is implemented as variability in the magnitude of each individual synaptic event.

The activity of the output layer of the network controls the direction of virtual entity's movement. Each of the output layer cells is mapped to a direction. The output layer cell (*O_j_*) that spikes the greatest number of times during the first half of an epoch defines the direction of movement on that epoch. If there is a tie the cell that spikes first determines direction. If no cells in the output layer fire the virtual entity continues in the direction it traveled during the previous epoch. There is 2% chance on every move that the virtual entity will ignore any output and instead move in a direction 45 degrees off of its direction on the last move. This random variability prevents infinite loops of virtual entity's motion during the learning process.

Plasticity in our model is based on a rewarded STDP paradigm [1], [2], [3], [4] implemented between layers *H* and *O*. A spike in a post-synaptic cell (*O_j_* of the output layer) that directly follows a spike in a pre-synaptic cell (*H_i_* of the hidden layer) creates a “*pre before post*” event. Additional post-synaptic spikes do not create additional *pre before post* STDP events. Likewise a spike in a pre-synaptic cell that directly follows a spike in a post-synaptic cell creates a “*post before pre*” event. Additional pre-synaptic spikes do not create additional *post before pre* STDP events.

The value of an STDP event, represented by *vE*, is calculated using the following equation [41], [83]:
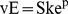





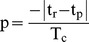
(1)Here *k* is equal to −0.025 in the case of a *post before pre* event and 0.025 in the case of a *pre before post* event. The variable *S* is the strength of the connection. *t_r_* and *t*_p_ are the times at which the pre and post synaptic spiking events occurred respectively. T_c_ is the time constant and is equal to 10 ms.

The STDP events are not immediately applied to the respective synapse *W_ij_* between neurons *H_i_* and *O_j_*. Instead they are stored as traces for later use. Each trace remains stored for 5 epochs after its creation and then is erased. If cases where the network sizes are larger than those described here the traces may be stored for a longer period. While still stored a STDP trace will have an effect whenever there is a rewarding or punishing event. If the network is rewarded or punished the change in synaptic strength of the synapse *W_ij_* is described as:
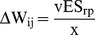





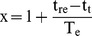
(2)Here *vE* is defined by equation(1). *S_rp_* is the scale of reward when the network is rewarded and the scale of punishment when the network is punished; *t_re_* is the time the reward or punishment occurred and 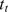 is the time the event trace was created; *T_e_* is the duration of an epoch.

The network is rewarded when the virtual entity moves to a “food” location. It is punished when it moves to a location without food.

The scale of reward is increased in inverse proportion to the sum of the cells outgoing synaptic strengths from hidden layer *H* to the output layer *O*:
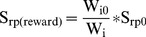
(3)Here 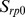 is constant value that corresponds to STDP strength and 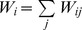 is a total synaptic strength of all connections from specific cell *H_i_* to all cells *O_j_* of the output layer. *W_io_* is a constant that is set to the value of *W_i_* at the at the beginning of the simulation. For the scale of punishment, 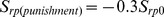. It remains constant and is not affected by the sum of the strength of the cells synaptic outputs. The effect of these rules is that the cells with lower total output strength increase their output strength more easily. We have found that creating competition between a cell's synaptic outputs by having the increased strength of one synapse affect the rate of strength increase of other synapses reduced the chance that a single cell middle layer cell would be capable of regularly causing action potentials in multiple output cells simultaneously. When a single middle layer cell caused multiple output layer spikes the spikes indicating the direction the entity did not move would be rewarded along with the one which indicated the direction the entity did move. This creates strong, stable, maladaptive connections.

To ensure that all the output neurons maintained a relatively constant long term firing rate, the model incorporated homeostatic synaptic scaling [76]. The total synaptic input 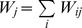 to a given output cell *O_j_* is set to be equal at each time step to the target synaptic input *W_j_ = W_j0_* - a slow variable that varies over many epochs and depends on the activity of that cell *O_j_* and activity of its pre-synaptic cells. If a cell *O_j_* repeatedly receives input but does not fire in response, the *W_j0_* is increased. If the cell responds with multiple spikes the *W_j0_* is gradually reduced. 
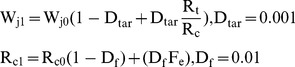
(4)Here 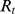 is the target rate of firing for the neuron in spikes per epoch and 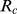 is the estimate of the cells current firing rate. 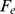
is the number of times the cell has fired this epoch. This update takes place every epoch (600 time steps).

To ensure that total synaptic input *W_j_* remains unaffected by plasticity events of individual connections at individual time steps and equal to *W_j0_*, we implemented scaling process that occurs after each STDP event. When any excitatory connection increases in strength, all the other excitatory connections incoming to that cell decrease in strength by a “*scale factor*” *S_f_* to keep *W_j_ = W_j0_*

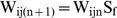
(5)Where 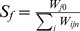, *W_ijn_* are synaptic weights right after STDP event but before scaling and *W_ij(n+1)_* are synaptic weights after scaling; *W_j0_* is from equation 3.

The model does not include mechanisms for inhibitory plasticity. All inhibitory connections *WI_ij_* incoming to cell *O_j_* from all cells *HI_i_* of the inhibitory layer have uniform strength. The sum of their inhibitory strength is held equal to the sum of the strength of all excitatory connections coming into the same cell. In other words at the each time step we scale *WI_ij_* so 
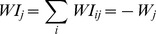
(6)A “hunger mechanism” is included that activates after an extended period of not receiving food. When activated it causes the virtual entity to ignore input layer activity and move in the last direction moved (98% probability) or change to a random new direction (2% probability). The behavior continues until it moves on to a food space. It is used to prevent the virtual entity from moving in infinite loops during the learning process.

### Map based neuronal models

To allow for efficient network simulations, we used a reduced model of a spiking neuron described by difference equations (map) [75], [84], [85]. The model is described by the following equations: 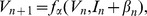 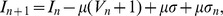 where 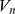 is the membrane voltage, 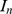 is a slow dynamical variable describing the effects of slow conductances, and 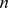 is a discrete time step (∼0.5 msec). Slow temporal evolution of 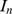 was achieved by using small values of the parameter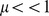. Input variables 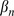 and 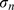 were used to incorporate external current 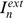 (e.g., synaptic input):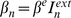, 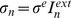. The nonlinearity 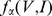 was designed in the form of a piece-wise continuous function:
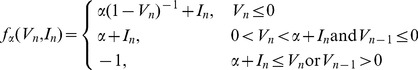
(7)To convert the dimensionless “membrane potential” *V* to the physiological membrane potential *V_ph_*, the following equation was applied: 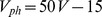
[mV] [76].

This model, despite its intrinsic low dimensionality, produces a rich repertoire of dynamics and is able to mimic the dynamics of Hodgkin-Huxley type neurons both at the single cell level and in the context of network dynamics [75], [85]. A fast spiking neuron model (Fig. 1) was implemented to simulate the neurons in the network.

To model synaptic interconnections, we used conventional first order kinetic models of synaptic conductances rewritten in the form of difference equations: 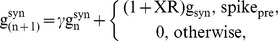
and the synaptic current computed as:
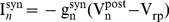
(8)Here *g_syn_* is the strength of synaptic coupling, and indices *pre* and *post* stand for the presynaptic and postsynaptic variables, respectively. The first condition, “spike*_pre_*”, is satisfied when presynaptic spikes are generated. Parameter *γ* controls the relaxation rate of synaptic conductance after a presynaptic spike is received (0≤γ<1). The parameter *R* is the coefficient of variability in synaptic release. The standard value of *R* is 0.16. *X* is a randomly generated number between -1 and 1. Parameter *V_rp_* defines the reversal potential and, therefore, the type of synapse: excitatory or inhibitory. A single IPSP produced in a postsynaptic excitatory cell by a spike in a presynaptic interneuron is shown in Fig. 1C. The term 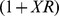 introduces a variability in synaptic release such that the effect of any synaptic interaction has an amplitude that is pulled from a flat distribution ranging from 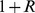 to 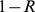 times the average value of the synapse.

### Reference strategies

In order to compare the performance of the network 4 automated methods of movement were designed. These strategies did not rely on the output of any network but used simple heuristics based on the location of food within the entities visual range (a 7 by 7 area centered on the entity).

Strategy 1 does not base the movement of the entity on the location of food. It initially selects a random direction to move. Each new epoch there is a 2% chance that the direction will change by 45 degrees (left or right chosen randomly). Otherwise it moves in the direction it moved on the previous epoch. In the standard 10% randomly distributed food environment it has an average success rate of 8.7% at obtaining food a given move.

Strategy 2 functions as strategy 1 with the exception that when food is in at least one square that is adjacent to the entity the next move will be to a square that contains food. This strategy has an average success rate of 32.3% under standard conditions.

Strategy 3 behaves as Strategy 1 only when no food is present within the visual field. When food is within the visual field the chosen move will be in the direction of one of the closest food. Which food is moved toward is chosen randomly if several food elements are at the same distance. Under standard conditions this strategy has an average success rate of 53.1%.

Strategy 4 is the most successful strategy. When no food is present in the visual field it behaves like Strategy 1. When food was present the strategy would search through all possible sets of 5 moves within the visual field. It then choses the set of moves that would result in the most food being obtained and makes the first move from that set. If multiple sets of moves obtain the same number of food the set which obtains food sooner is preferred. If multiple sets have the same sequence of food being obtained one of those sets is chosen randomly. Under standard conditions this strategy has an average success rate of 56.0%
